# Physical constraints lead to parallel evolution of micro- and nanostructures of animal adhesive pads: a review

**DOI:** 10.3762/bjnano.12.57

**Published:** 2021-07-15

**Authors:** Thies H Büscher, Stanislav N Gorb

**Affiliations:** 1 Department of Functional Morphology and Biomechanics, Institute of Zoology, Kiel University, Am Botanischen Garten 9, 24118 Kiel, Germany

**Keywords:** adhesion, attachment devices, biomechanics, convergence, friction, substrate compliance

## Abstract

Adhesive pads are functional systems with specific micro- and nanostructures which evolved as a response to specific environmental conditions and therefore exhibit convergent traits. The functional constraints that shape systems for the attachment to a surface are general requirements. Different strategies to solve similar problems often follow similar physical principles, hence, the morphology of attachment devices is affected by physical constraints. This resulted in two main types of attachment devices in animals: hairy and smooth. They differ in morphology and ultrastructure but achieve mechanical adaptation to substrates with different roughness and maximise the actual contact area with them. Species-specific environmental surface conditions resulted in different solutions for the specific ecological surroundings of different animals. As the conditions are similar in discrete environments unrelated to the group of animals, the micro- and nanostructural adaptations of the attachment systems of different animal groups reveal similar mechanisms. Consequently, similar attachment organs evolved in a convergent manner and different attachment solutions can occur within closely related lineages. In this review, we present a summary of the literature on structural and functional principles of attachment pads with a special focus on insects, describe micro- and nanostructures, surface patterns, origin of different pads and their evolution, discuss the material properties (elasticity, viscoelasticity, adhesion, friction) and basic physical forces contributing to adhesion, show the influence of different factors, such as substrate roughness and pad stiffness, on contact forces, and review the chemical composition of pad fluids, which is an important component of an adhesive function. Attachment systems are omnipresent in animals. We show parallel evolution of attachment structures on micro- and nanoscales at different phylogenetic levels, focus on insects as the largest animal group on earth, and subsequently zoom into the attachment pads of the stick and leaf insects (Phasmatodea) to explore convergent evolution of attachment pads at even smaller scales. Since convergent events might be potentially interesting for engineers as a kind of optimal solution by nature, the biomimetic implications of the discussed results are briefly presented.

## Review

### Animal attachment systems

**Figure 1:**
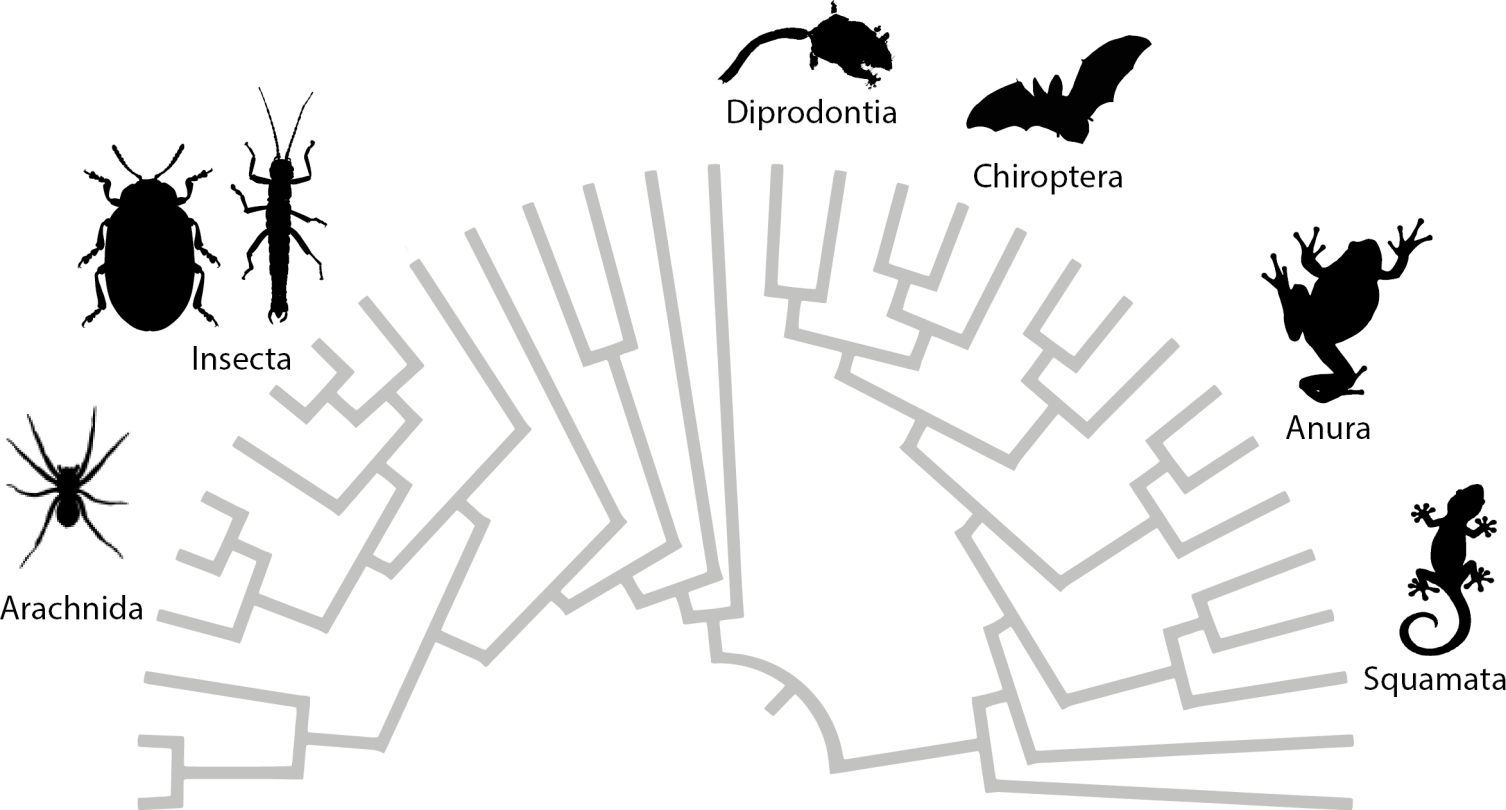
Diversity of investigated study organisms with attachment devices visualised across the animal tree of life. Attachment devices are almost omnipresent in animals.

Attachment is of major importance in the biology of most living animals. Secure attachment to specific surfaces is essential for many animals, for example, to maintain access to nutrients and to support locomotion on any terrain that necessitates adhesion to the substrate. The properties of the specific surfaces in their natural environments shaped the morphology and function of the attachment devices of the animals. The characteristics of similar habitats resulted in similar selective pressures for various different animal groups. Attachment devices are omnipresent in animals (Figure 1), especially for terrestrial locomotion. The morphological and ultrastructural backgrounds on the ability of animals to attach to and walk on vertical surfaces and ceilings have been studied in detail in many animal taxa, including insects [1–4], arachnids [5–9], tree frogs [10–11], arboreal salamanders [12], lizards [13–21], Echinodermata [22–24], and Mammalia [25–32]. These studies show that during the course of biological evolution, animals have developed in a convergent manner two distinct types of structures to attach themselves to a variety of substrates: hairy (setose) pads and smooth pads. Next, we will focus on the attachment systems used for terrestrial locomotion.

**Figure 2:**
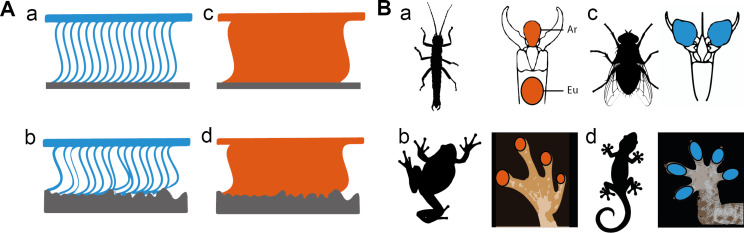
Attachment systems of animals. (A) Schematic representation of hairy (a, b) and smooth (c, d) attachment pads. Both systems provide adjustment to the surface profile of smooth (a, c) and corrugated (b, d) substrates. (B) Examples of smooth and hairy adhesive systems. (a) Smooth arolia (Ar) and euplantulae (Eu) in stick insects. (b) Smooth toe pads in frogs. (c) Hairy pulvilli in flies. (d) Hairy toe pads in geckos. Colours correspond to the type of attachment pad (cyan = hairy; orange = smooth). Figure 2A and the tarsus drawings in 2Ba and 2Bc were adapted with permission from [1], R. G. Beutel et al., “Ultrastructure of attachment specializations of hexapods (Arthropoda): evolutionary patterns inferred from a revised ordinal phylogeny”, J. Zool. Syst. Evol. Res., with permission from John Wiley and Sons. Copyright © 2001 John Wiley & Sons. This content is not subject to CC BY 4.0.

Hairy pads are covered with setae, acanthae and microtrichia [33], fine cuticular surface outgrowths which, due to their small size and flexibility, can maximise the extent of contact with a wide range of microscopically rough substrate profiles (Figure 2). Also, due to the low bending stiffness of their terminal plates, can even adapt to substrates with roughness on a sub-nanometre scale [1,3–4,34]. Smooth pads can also maximise their contact areas with a variety of substrates due to their specialised material structure and properties at micro- and nanoscales [35]. Interestingly, in the course of the biological evolution, both functional solutions independently appeared many times in different animal groups.

### Diversity of insects and their attachment devices

Represented by more than one million described species, insects constitute the majority of animals on earth. With their astonishing diversity, they are one of the most remarkable lineages in the 3.5 billion years of life history on this planet [36]. Insects are, in terms of diversity, biomass, and organismic interactions, undisputedly one of the most important groups of animals [37]. The chitinous exoskeleton is often mentioned as the basis of structural diversification and considered a key innovation for the success of insects [37–39]. The versatility of the cuticular integument provides a broad array of tools for various functional demands [3,40]. Specifically, the wings are considered important to facilitate mobility, dispersal, and escape from predators [41–46]. Furthermore, the ability to efficiently move in different environments promotes niche diversity and subsequently species diversity in insects [47]. A key feature for mobility, next to the evolution of wings, is the evolution of a segmented leg in arthropods [48]. These paired, articulated appendages, in combination with the hardened exoskeleton, served for both Arthropoda and insects, in particular, as a tool to become ubiquitous in nearly all habitats on earth [49–51]. Besides exploiting the advantages of tagmosis, the adaptability of the jointed limb enabled settlement in different habitats and exposed numerous opportunities for adaptive radiation [39].

The legs of insects are usually used for walking and are adapted to locomotion across different terrains [39,52]. Even without morphological specialities, like jumping or digging, legged motion is very diverse. Some groups specialized in their micro- and nanostructures towards very specific substrates: Water striders, for example, run on the water surface [53–55] and ectoparasitic flies are highly modified to remain attached to their hosts and move on them [56]. There are numerous other functional modifications on insect legs, including silk production (e.g., [57]) or prey capturing [58], but one is of major importance for nearly all insects: the attachment system.

During the evolution of insects, two factors have been suggested to be predominantly influence the evolution of attachment devices. Wings enabled the dispersal and colonization of different environments, and flying forced insects to land and attach to several different, often unpredictable, substrates [1–2,59–60]. Many insects are phytophagous, often strongly focused on a narrow spectra of angiosperm plants [37,61–66]. Additionally, plants are not only used for nutrition but also represent sites for foraging, mating, and placement of the offspring [67–71]. Hence, the evolution of the attachment systems appears to be associated with the coexistence of insects and plants.

The coevolution of angiosperms and insects is suggested to have resulted in an extensive adaptive co-radiation [72–74]. Besides chemical weapons against herbivorous insects, a large variety of surface micro- and nanostructures evolved on the plants. As a response, the insects developed different micro- and nanostructures to walk on those surfaces and attach to them [1]. Consequently, a plethora of different attachment devices has been evolved in insects.

**Figure 3:**
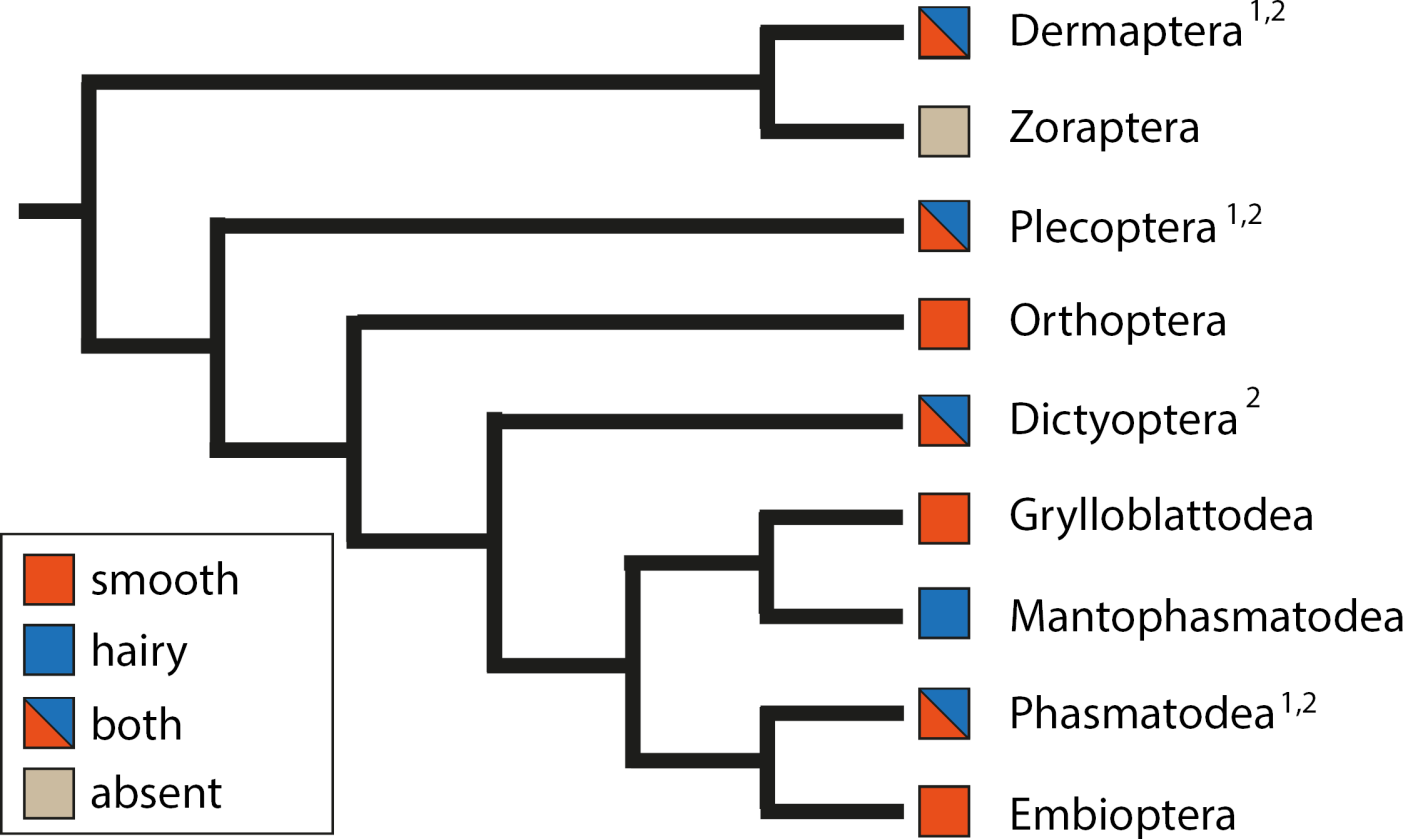
Phylogeny of Polyneoptera (following [122]). Coloured squares indicate the type of attachment pads, ^1^different pad types on the same tarsus, and ^2^in different species.

Although insects are extremely diverse, the design of attachment devices within insects can be subdivided into the two fundamental types of hairy and smooth attachment pads [1,3], as abovementioned, similarly at the level of the animal kingdom. In insects, the hairy structures consist of deformable adhesive setae, typically originating from the tarsus itself (Figure 2) and occur in different groups of insects. They are common in Coleoptera (e.g., [75–95]), Dermaptera (e.g., [96]), Megaloptera [1,97–98], Strepsiptera [1,99], Mantophasmatodea [2,59], and Diptera (e.g., [56,60,100–107]). In stick insects (Phasmatodea) [108–109] and true bugs (Heteroptera) [90,110–121], some species are reported to have hairy attachment structures, although the majority of the taxa have smooth ones (Figure 3). Although very similar in shape and identical in function, these micro- and nanostructures evolved in a convergent manner in different insect groups.

Smooth attachment systems, on the other hand, comprise soft cuticular pads without elongated fibrillar outgrowths. Usually, such attachment pads, similarly to the hairy ones, are ventrally located on the tarsus (e.g., euplantulae) or at the pretasus (e.g., single arolia or paired pulvilli). In some cases, attachment structures are present on the tibia as well [1,66,109,123–125]. Smooth attachment pads are found in most groups of insects, for example, in Orthoptera [126–133], Siphonaptera [1], Phthiraptera [1,134], Mantodea [1,135], Hymenoptera [1,136–148], Embioptera [109,149–150], Ephemeroptera, in form of a claw pad, [1], Thysanoptera [1,151–153], Blattodea [154–156], Phasmatodea [2,59,108–109,157–164], Stenorrhyncha [66,123–124,165–166], Auchennorrhyncha [167–170], and some Mecoptera [1,103,171].

Yet in many groups, the type of attachment system is not necessarily uniform throughout the entire group (e.g., Phasmatodea, [109]). Unfortunately, broad comparative analyses based on several species per group are missing for most insect lineages. In addition, the same anatomical structure might be hairy or smooth in different representatives of the same group (e.g., the pulvilli of flies [60] or the plantulae of Hymenoptera [146]) which are hairy in some taxa and smooth in others. Similar structures in different orders (e.g., the pulvilli of flies and true bugs [1,60]) or the plantulae in Hymenoptera (e.g., [172]) and euplantulae in other insects (e.g., [173]), can be either hairy or smooth in different groups. Consequently, the majority of these attachment structures are most probably not homologous, but independently evolved multiple times [1,146,174–175]. In Acercaria (Psocodea, Hemiptera, and Thysanptera, according to Börner [176]), the pulvillus independently evolved at least two times [118]. In some groups, a combination of the two types is found on the tarsus (e.g., smooth arolia and hairy soles or hairy euplantulae in Mantophasmatodea [2,59], Tipulidae (Diptera) [1,60,177–178], Plecoptera [1,179] and Lepidoptera [1,180–183]).

### Parallel evolution: exemplified by Polyneoptera

The Polyneoptera are a group of insects which comprises around 40.000 extant species and includes the majority of the hemimetabolous insects [122]. Although the number of taxa is much less than in other groups of insects, like Diptera or Coleoptera, many details of their evolution, such as changes in morphology, behaviour, or lifestyle remain unresolved. One reason lies in the notable differences in the subgroups of Polyneoptera and their strong ecological differentiation, impeding a reliable reconstruction of the internal relationships for many years [122,184–185]. The attachment pad morphology is both an indicator and a result of the complex mesh of adaptations. The presence of hairy and smooth attachment structures within Polyneoptera is shaped by convergence on different levels (Figure 3). While adhesive structures are absent and probably secondarily reduced in Zoraptera [59], several other groups include hairy structures. Adhesive hairs not only independently evolved in Dermaptera [96], Plecoptera [179], Phasmatodea [108–109], Dictyoptera [154], and Mantophasmatodea [2,59], but also reveal different stages of reversals or repetitive origins within these. While all mantophasmids possess hairy euplantulae and in Dictyoptera, Plecoptera, and Phasmatodea only very few species possess hairy attachment structures, the hairy structures just within Dermaptera independently evolved multiple times [96]. Polyneoptera are not only striking examples of the convergent presence of the primary types of attachment structures, but additionally reveal functional micro- and nanostructures on the adhesive devices of many groups [133].

Smooth attachment pads are not always absolutely smooth. Mostly they bear surface microstructures with certain functions [133]. In many polyneopteran species the attachment pads have been described to be smooth; however, they are covered with cuticular patterns or protuberances [133]. These outgrowths have been differentiated from setae/acantae according to their low aspect ratio (height-to-width ratio). In contrast to hairy (seta-like) protuberances with high aspect ratios (higher than 10), many species exhibit smaller sized cuticular nubs (aspect ratio usually <5). Nubs and other surface patterns are reported to be adaptations to tune the contact formation of smooth attachment pads towards specific substrate conditions [133,158,160,162]. Similar attachment microstructures (AMS) are found, in a convergent manner, in different polyneopteran groups, in species with a similar ecology [108–109,133,161,163]. In general, due to the lack of broad comparative studies on many taxa with smooth pads, the distribution of different AMS within Polyneoptera is not well resolved. However, stick and leaf insects (Phasmatodea) arose as a fascinating model group for answering attachment-related evolutionary questions.

### Stick insects and their adhesive systems

**Figure 4:**
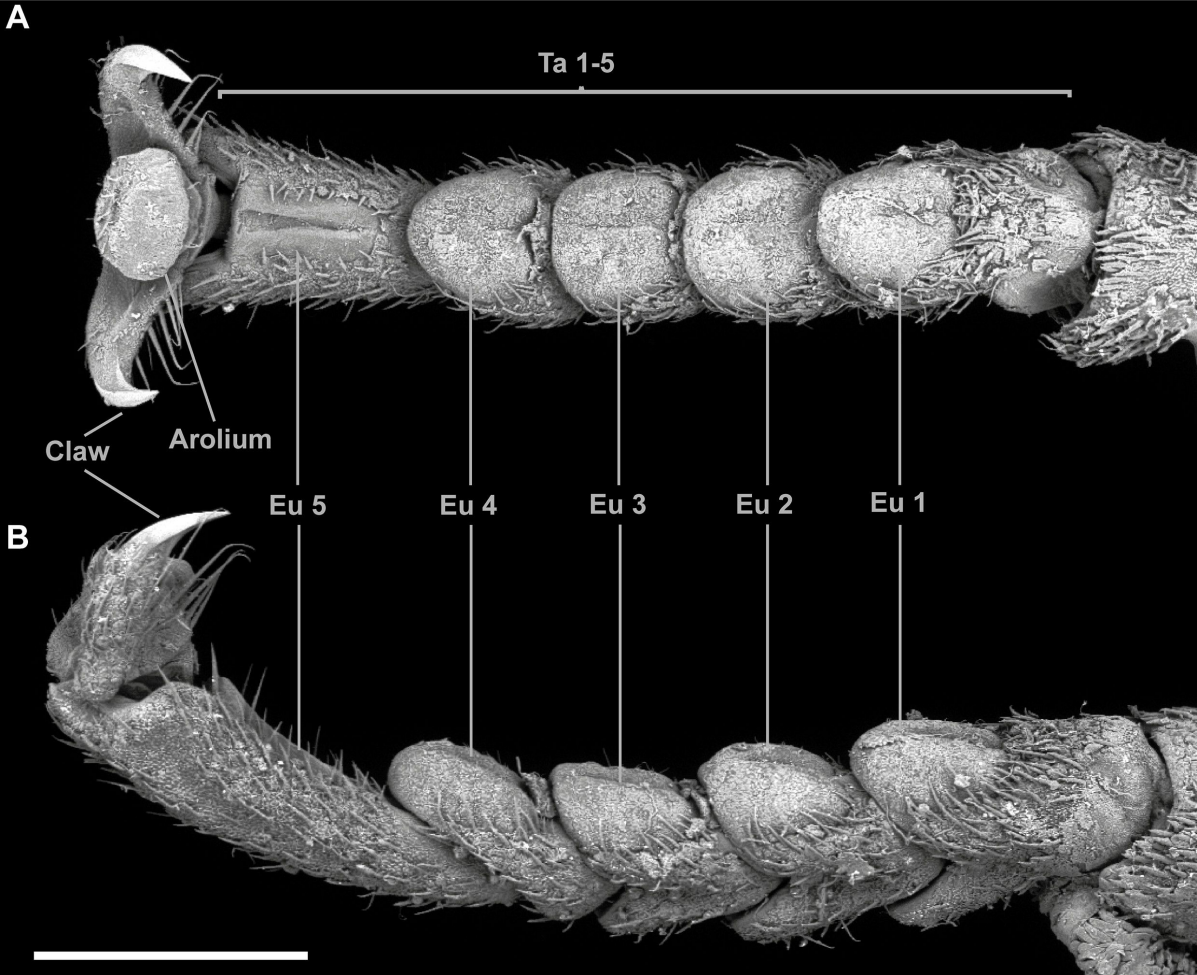
Scanning electron microscopy (SEM) images of a typical phasmatodean tarsus. *Orestes draegeri* Bresseel & Constant, 2018. (A) Ventral view. (B) Lateral view. Ta1–5, tarsomeres; Eu1–5, euplantulae; Cl, claw; Ar, arolium. Scale bars: 1 mm.

Stick and leaf insects are an impressive model group for the exploration of many evolutionary aspects, especially convergence. Limited spatial dispersion and extensive adaptive radiation led to a high degree of convergent traits in Phasmatodea, (e.g., in terms of visual camouflage [184–187], oviposition techniques [109,188–194], different degrees of wing loss [195–197], and ecomorphs (morphological forms with similar ecological occupancy) with specific vertical stratification within the vegetation [108,198–199]). Phasmids are predominantly nocturnal insects that are distributed nearly worldwide and exceptionally herbivorous [184–187,194,200], with the highest diversity in the tropics. The majority inhabits shrubs and trees of the most tropical and temperate ecosystems [201–202]. As suggested by their name, many stick and leaf insects are impressively well-camouflaged in these environments and visually blend with their surroundings due to their outer appearance [184,186–187,194,203]. This visual camouflage evolved prior to the emergence of angiosperms, when gymnosperms represented the majority of plant diversity [204–205]. The oldest fossil record for stick insects dates back to 165 mya (Jurassic) and already revealed morphological specializations to enable mimicry [205]. Another described fossil stick insect from the Cretaceous, *Cretophasmomima melanogramma* [204], already impressively copies the visual appearance of plants. Its tegminal coloration visually mimics characteristics of the gymnosperm *Membranifolia admirabilis* Sun and Zheng, 2001, found as a common part of the Cretaceous flora of the same formation [204]. Subsequently, phasmids and plants probably co-radiated when stick insects started imitating their floral surroundings to avoid predators [206–207]. During the emergence of angiosperms, and their major radiation [208–209], stick insects evolved in a similar pace [194,198,207,210–211], possibly in response to the burgeoning diversity of plants and corresponding adaptations [194,212–213]. This not only resulted in a strong host-specific mimicry response for many recent phasmids, but also led to several counter-adaptations against herbivory on the plant side (e.g., [214–217]). While plants evolved defence strategies to repel the herbivorous stick insects, the latter evolved strategies to overcome the strategies developed by the plants [218–224]. The ongoing arms race between stick insects and their host plants caused adaptations in the attachment system of phasmids to several different surfaces [108–109,158,161–163], which in turn resulted from the strong association of plants and herbivorous insects. A typical tarsus of Phasmatodea (Figure 4) consists of five tarsomeres. It is equipped with two claws and an arolium on the pretarsus, as well as euplantulae on the proximal four to five tarsomeres [1–2,109,161–162,225]. Except for the euplantulae of some Aschiphasmatini (Aschiphasmatinae) that are covered with adhesive setae [109], all attachment pads of Phasmatodea are smooth; however, they are covered with functional surface microstructures. Although the arolia are rather uniform in their morphology, their surface microstructure reflects the basal sister-group relationship of *Timema* and Euphasmatodea (all remaining Phasmatodea). The arolia of *Timema* are covered with acanthae (unicellular cuticular outgrowths (according to [33]), those of Euphasmatodea are completely smooth [1–2]. The euplantulae (i.e., the proximal tarsal attachment pads) reveal a high diversity of AMS, which is suggested to be of functional relevance in terms of adaption to surfaces found in the species-specific environments [1–2,108–109,158,163].

**Figure 5:**
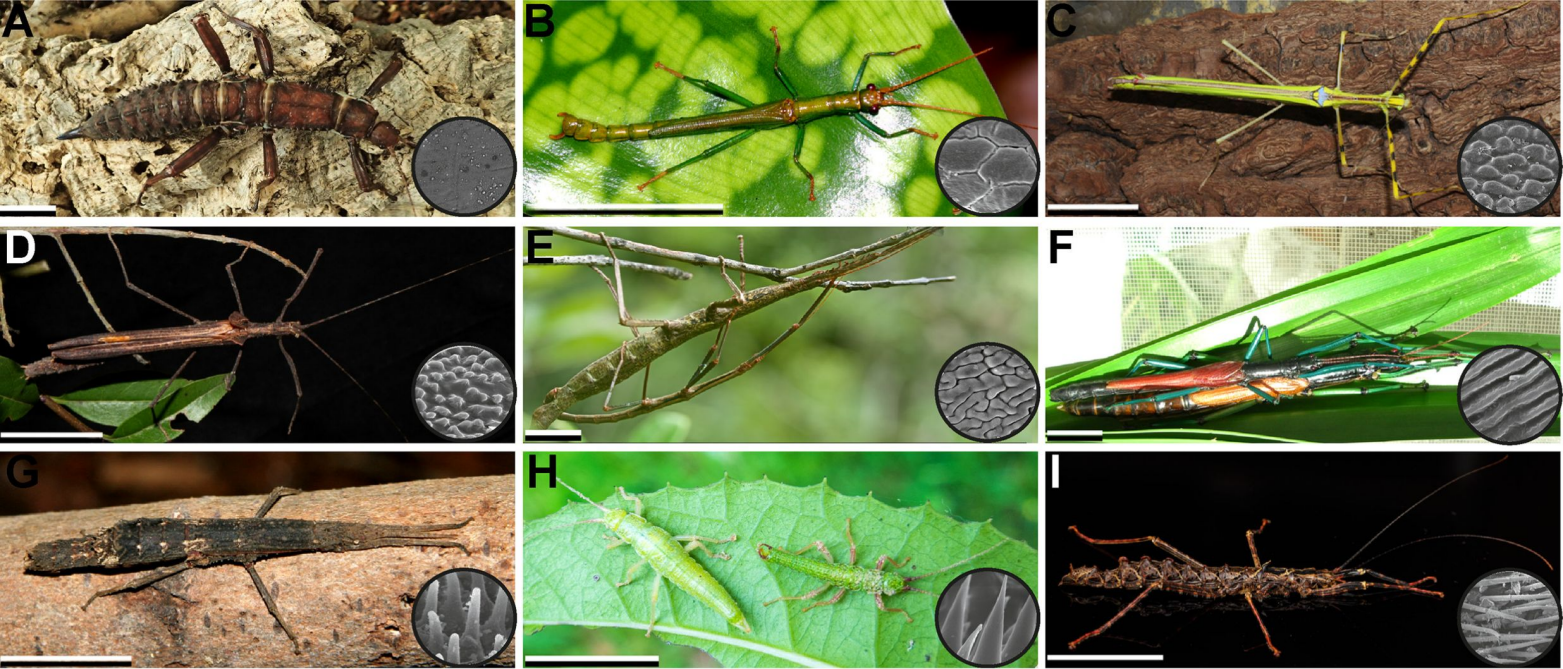
Diversity of stick insect ecomorphs and their respective euplantular AMS. (A) *Eurycantha calcarata*, female, smooth. (B) *Dajaca monilicornis*, male, hexagonal. (C) *Anarchodes annulipes*, female, flat pads. (D) *Pseudophasma velutinum*, female, small nubs. (E) *Leiophasma* sp., couple, maze. (F) *Megacrania phelaus*, couple, ridges. (G) *Orestes mouhotii*, female, long nubs. (H) *Timema* sp., couple, acanthae, image provided by S. Büsse. I. *Dinophasma saginatum*, female, hairy. Scale bars: 1 cm. Figure 5A–I were adapted from [108] (© 2018 Büscher et al., published by Frontiers, distributed under the terms of the Creative Commons Attribution License (CC BY)).

A comparative study of a large number of stick insect species yielded twelve different types of AMS on the euplantulae (Figure 5), including one lineage with adhesive setae on the euplantulae [109]. Previous studies hypothesized a phylogenetic signal of characters of the tarsal attachment system. Those were discussed for the placement of Phasmatodea within insects [1–2].

Other studies tried to make use of AMS for the reconstruction of the internal systematics of Phasmatodea [159,161,226]. These studies revealed distinctive features above the species level in the form of AMS of the euplantulae. However, these features apparently represent indications of similar habitats and not of phylogenetic relationships, as suggested by the character mapping based on molecular data [108].

The distribution of the euplantular AMS suggests a high dependence of the microstructure to the habitat of the species. Ground-dwelling stick insects reveal nubby microstructures unrelated to their phylogenetic position, as well as canopy-dwelling species, which possess smooth structures without cuticular patterns on the surface [108]. The various types of AMS within phasmids stand out in comparison to other insects, judging by the diversity of attachment structures reported in the literature [60,96,179]. The high degree of convergence in the AMS of phasmids probably indicates adaptations to the surfaces encountered in the environment [108], as hypothesized in the literature [108–109,158,161–163]. The disparity of AMS among the phylogenetic relationships, however, does not reveal a clear clustering of species with the same AMS as suggested by previous authors [1,159–161,225]. The convergent presence of the same microstructures, in contrast, is a result of similar demands for adhesion in the respective habitats, which means that the physical rules of contact mechanics have a very strong influence on the adaptive evolution of the attachment structures in general. The reason is that similar AMS provide similar properties to optimize the attachment to particular surfaces. Different AMS follow specific functional principles which are beneficial in specific environments and, therefore, occur in a convergent manner within phasmids with similar eco-morphological demands.

### Facilitation of rapid parallel evolution

Stick and leaf insects obviously evolved in close connection to the evolution of plants [204]. The high diversity of AMS indicates a potential for rapid evolution. The versatile solutions for different attachment problems evolved in a convergent manner [109]. As the degree of convergence in the AMS is high and different AMS types coexist in the same groups, the adaptation to the corresponding natural surfaces probably took place in a comparatively short period of time [163]. Using a mathematical model, the potential of self-assembly of the structures observed in the AMS of phasmids have been recently evaluated based on a reaction–diffusion model considering a two-morphogen interaction. The self-formation of different patterns in nature can be explained by the reaction–diffusion model proposed by Alan Turing [227]. This model has been previously employed to model similar patterns on insects as well to investigate evolutionary scenarios (e.g., the patterns of nanocoatings on the corneae of different lineages [228]). Employing this mathematical model to access self-formation and transformations of the euplantular AMS of phasmids yielded the prediction of stable patterns of functional AMS on the euplantulae of phasmids. The transitions observed in the simulations were used to evaluate the adaptability of the structures, transitions between the structural patterns which could reflect the evolutionary processes, to re-evaluate the potential ancestral state of stick insect AMS, and to suggest a rapid response and versatile adaptability of the AMS in a relatively short evolutionary time [163]. The Turing model indicates a fast response when animals face changes in surface composition, contributing to a flexible adaptability of the functionalized attachment surfaces. Similar changes in the surface geometry of functional microstructures have already been shown to arise within less than 5000 years [229–230]. The ecomorphological specialization, influenced by the ability of the insects to securely attach to the surface of a specific plant, contributes to the specialization of insects to plants [231]. Nevertheless, this also plays a role in host fidelity and potentially even speciation. Although the acquisition of flight supposedly has induced the diversification of attachment structures in insects (e.g., [1]), it is likely that in phasmids the convergent loss of flight ability [195,225] enhanced the host–plant dependence. The adaptation to specific plant surfaces due to a strong coevolution with plants enhances diversity even more. This resulted in frequent independent origins of the same AMS in different lineages of phasmids, and aided to the achievement of the demanded adhesive properties in their respective environments. Consequently, the convergent presence of the same AMS is primarily a result of the same environmental condition found and to establish the necessary functional principle.

### Functional principles

Studies on different groups of insects have shown that claws generally contribute to the attachment on rough surfaces due to friction and mechanical interlocking [83,89,92,232–236]. The performance of claws depends on the radius of the claw tip in relation to the curvature of the surface irregularities [83,234,237–238]. However, in combination with the claws, the attachment pads provide adhesion to surfaces with different roughness conditions [162,238]. This ability has numerous contact mechanical demands (called below as “functional principles”), which evolved under similar boundary conditions in different groups and hence reveal convergent results. In the next subsections we discuss the following functional principles: (1) Adaptation to fractal substrate surfaces due to hierarchical organization and thin surface layer, (2) micro- and nanostructural surface pattern and contact splitting, (3) pad material (structure) that is soft under compression but strong under tension, (4) anisotropy in fibre orientation, and (5) presence of fluid in the contact area.

#### Adaptation to fractal substrate surfaces due to hierarchical organization

**Figure 6:**
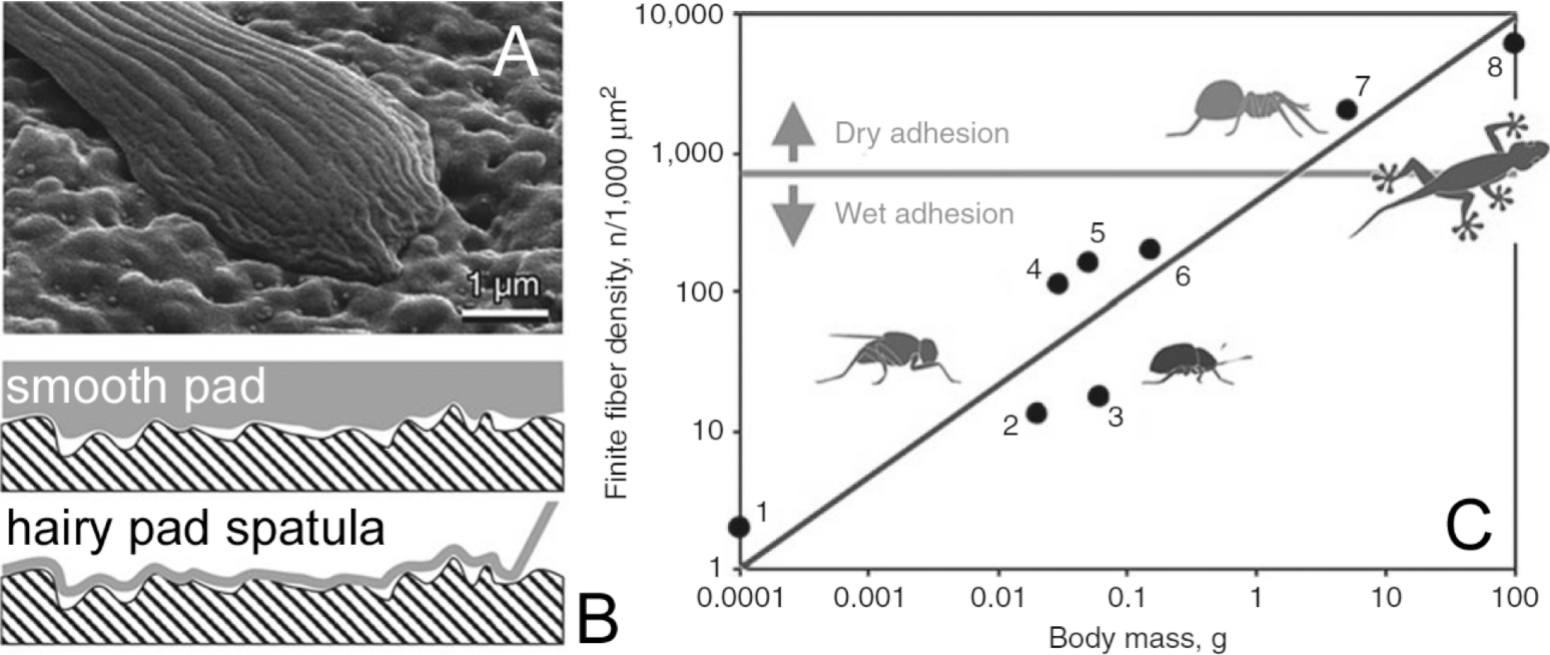
Compliancy of adhesive structures to the substrate (A,B) and contact splitting (C). (A) Contact of a spatula of the beetle *Gastrophysa viridula* with a surface with microscale roughness. (B) Soft smooth pad requires additional load to form an adhesive contact (B, upper image), whereas the adhesion interaction pulls the elastic thin film of the spatula into a complete contact with the rough substrate surface (Figure 6A and 6B are from [241] and were adapted by permission from Springer Nature from “Biological fibrillar adhesives: functional principles and biomimetic applications. In: Handbook of Adhesion Technology” by S. N. Gorb, Copyright 2011 Springer Nature. This content is not subject to CC BY 4.0). (C) Dependence of the contact density of terminal contacts on the body mass in fibrillar pad systems in representatives of diverse animal groups: 1, 2, 4, 5, flies; 3 beetle; 6 bug; 7 spider; 8 Gekkonid lizard (Figure 6C is from [247] and was adapted by permission from Springer Nature from “Biological Micro– and Nanotribology: Nature’s Solutions” by M. Scherge and S. N. Gorb, Copyright 2001 Springer Nature. This content is not subject to CC BY 4.0). The systems, situated above the solid horizontal line, preferably rely on van der Waals forces (dry adhesion), whereas the rest rely mostly on capillary and viscous forces (wet adhesion).

Hairs with high aspect ratios in the hairy systems and internal fibres/filaments of smooth systems bend during contact formation with the substrate (Figure 6A and Figure 6B). The pad can, therefore, work as a damper at high-speed deformations during jumping or landing. More importantly, in terms of contact mechanics, deformability functions as a basis for replicating a complex substrate profile during contact formation. Also, the hair- or rod-like organization of the pad architecture would allow an independent local load distribution over the area of contact between the pad and the substrate. This aids to the enhancement of the adaptation of the pad to the surface irregularities of non-smooth natural substrata. However, the convergent basic architecture of the pads can be tuned into different needs and constraints in the evolution of individual animal groups and species. The structural principle based on branching rods in smooth pads may, for example, additionally contribute to holding the shape of the pad.

An important structural feature of both types of attachment pads is the presence of a micro- or even nanoscopic superficial thin film contributing to the compliance of hairy and smooth attachment devices. In smooth systems the epicuticle covers the fibrous material of the pad and spatula, terminating tips of the cuticle outgrowths in hairy systems form the superficial film. These films are responsible for proper contact formation with the substrate due to their low bending stiffness at a minimum load [239]. The film/spatula is able to adapt to the surface profile and to replicate surface irregularities of certain length scales. The range of length scales to which the adaptation is possible depends on the stiffness of the film. Spatulae are able to adapt even to nanoscale roughness [240–241]. Thick films within the smooth pads of the bush cricket *Tettigonia viridissima* and the locust *Locusta migratoria* [129,132] adapt to the microscale roughness. However, the latter species has a lower adaptability to the surface roughness because of the much thicker superficial film than that of the previous species. In smooth pads, film terminating fibres, which are sometimes of an extreme high aspect ratio, prevent the lateral collapse (condensation, conglutination) of fibres [242–244]. They would otherwise condensate with each other and not work as separate springs [245]. The film also delimits the smooth pad as a reservoir filled with fluid and, under certain pressure, holds the pad as a stable unit [35,246]. The thicker superficial film in the desert species may also minimize water loss [132] and, presumably, it prevents the fragile fibrous material from wearing out during walking [126–127]. However, in thicker/stiffer films, the adhesive properties are, at the same time, reduced due to the reduced ability to form a close contact with rough substrata.

#### Surface pattern and contact splitting

The function of hairs/setae in hairy pads is partially discussed in the previous paragraphs. Comparative studies on different animal groups comprising hairy attachment pads reveal correlations between the morphometric features of the setal tips and the weight of these animals (Figure 6C): heavier animals possess smaller terminal contact elements, which are also more densely packed [247]. Contact splitting can be used to explain this scaling effect: following this principle, the adhesion on flat substrates can be increased by splitting the contact with the substrate into finer subcontacts [248–249]. As the scaling rules of mass and adhesive pad surface area are different, the area of the attachment devices cannot increase proportionally to the body weight of the animal Therefore, hairy systems increase the attachment strength by increasing the hair density and, consequently, increasing the amount of single contacts. This trend, however, differs for multiple reasons among different lineages in which hairy adhesive pads evolved in a convergent manner [250]. The small effective elastic modulus on the surface of hairy attachment pads resulting from contact splitting is fundamental to the adhesion on rough substrates [251] and contributes to an increased real contact area compared to unstructured materials under the same applied load. In general, based on broad comparative analyses, animal lineages that make use of dry adhesions (squamates and arachnids) seem to have more and smaller terminal contact elements than animals that rely on wet adhesions (insects).

Smooth attachment pads, without macroscopic hairs, are not ideally smooth in most cases, but rather wrinkled or, in some cases, patterned at the micrometre or submicrometre levels [35,109]. The upper sides of surface patterns in contact with a substrate may approach the counterpart very closely. In this case, solid–solid interactions occur between the pad material and the substrate. Under a certain load, a fluid is released out of the contact into the gaps between the outgrowths. The non-ideal smooth surface of the pad, similar to a tyre profile, prevents aquaplaning and enhances solid–solid interactions which are not only important for adhesion enhancement, due to van der Waals forces, but also for friction enhancement [252]. The fluid trapped by the gaps might be additionally used in the next step cycle. The prevention of aquaplaning is especially important for walking on wet surfaces in a rain forest environment or in temperate areas. Also, nubby pad microstructures can generate additional frictional grip on rough surfaces [158,162].

#### Pad material (structure) that is soft under compression but strong under tension

**Figure 7:**
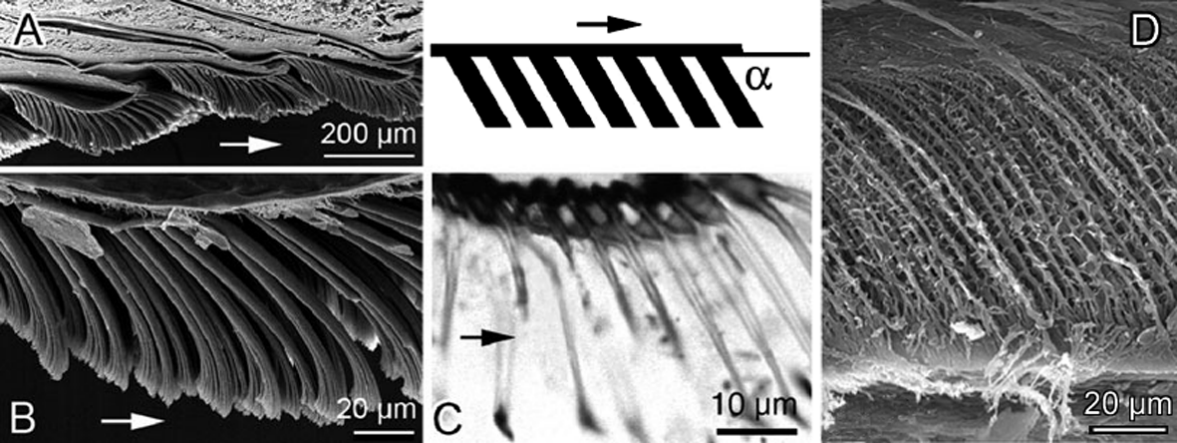
Convergent evolution of an asymmetry of micro- and nanostructural features (scheme is given in the inset) in animal attachment pads, leading to functional anisotropy upon contact. Longitudinal sections of pads are visualised in SEM (A,B,D) and in light microscope (C). (A,B) Tokay *Gekko gecko* (hairy pad). (C) Fly *Calliphora vicina* (hairy pad). (D) Bush cricket *Tettigonia viridissima* (smooth pad). The arrows indicate distal directions in all pads. Figure 7A–C is from [241] and was adapted by permission from Springer Nature from “Biological fibrillar adhesives: functional principles and biomimetic applications. In: Handbook of Adhesion Technology” by S. N. Gorb, Copyright 2011 Springer Nature. This content is not subject to CC BY 4.0. Figure 7D was adapted from [129] with permission from The Royal Society (U.K.), from Proc. R. Soc. B, S. N. Gorb, vol. 267, issue 1449, Copyright 2000; permission conveyed through Copyright Clearance Center, Inc.. This content is not subject to CC BY 4.0.

It is well known that an array of thin fibres is soft under compression, but exceptionally strong under tension [253]. This is the key principle of both pad architectures: hairy and smooth (Figure 7). The specific external (hairs/setae) or internal (fibres/foam) structures of the attachment pads are not only responsible for their softness under compression, but also for their stiffness under tension. The specific arrangement of thin hairs or fibres in the direction of tensile forces, acting on the pad in contact, aids in resisting such forces when the animal is hanging on a ceiling or wall, or sliding along the substrate. The relatively high tensile strength of a soft material would not be possible without such a fibre-like reinforcement. The fibrillar organisation of smooth pads represents their main structure/functional similarity to hairy pads.

#### Anisotropy in fibre orientation

Since fibres are normally not oriented perpendicularly to the pad surface (Figure 7), but rather at some angle (45–60°) and sloped into the distal direction, they do not buckle but rather bend under load, which makes the pad material even more flexible. The structural anisotropy of the pad material is also responsible for the frictional anisotropy [129]. The friction is higher while the pad is sliding in a proximal direction because the fibres of smooth pads or hairs of hairy pads can be more easily recruited in this case. Such a mechanism may secure a stable position of an animal on a ceiling. As the shear forces are applied proximally towards the body in this situation, and because of a stronger friction in this direction due to an intimate contact between the membranes/spatulae, the pad sliding can be prevented. The fibre anisotropy may also be involved in the detachment mechanism of the pad [106].

#### Presence of fluid in the contact area

**Figure 8:**
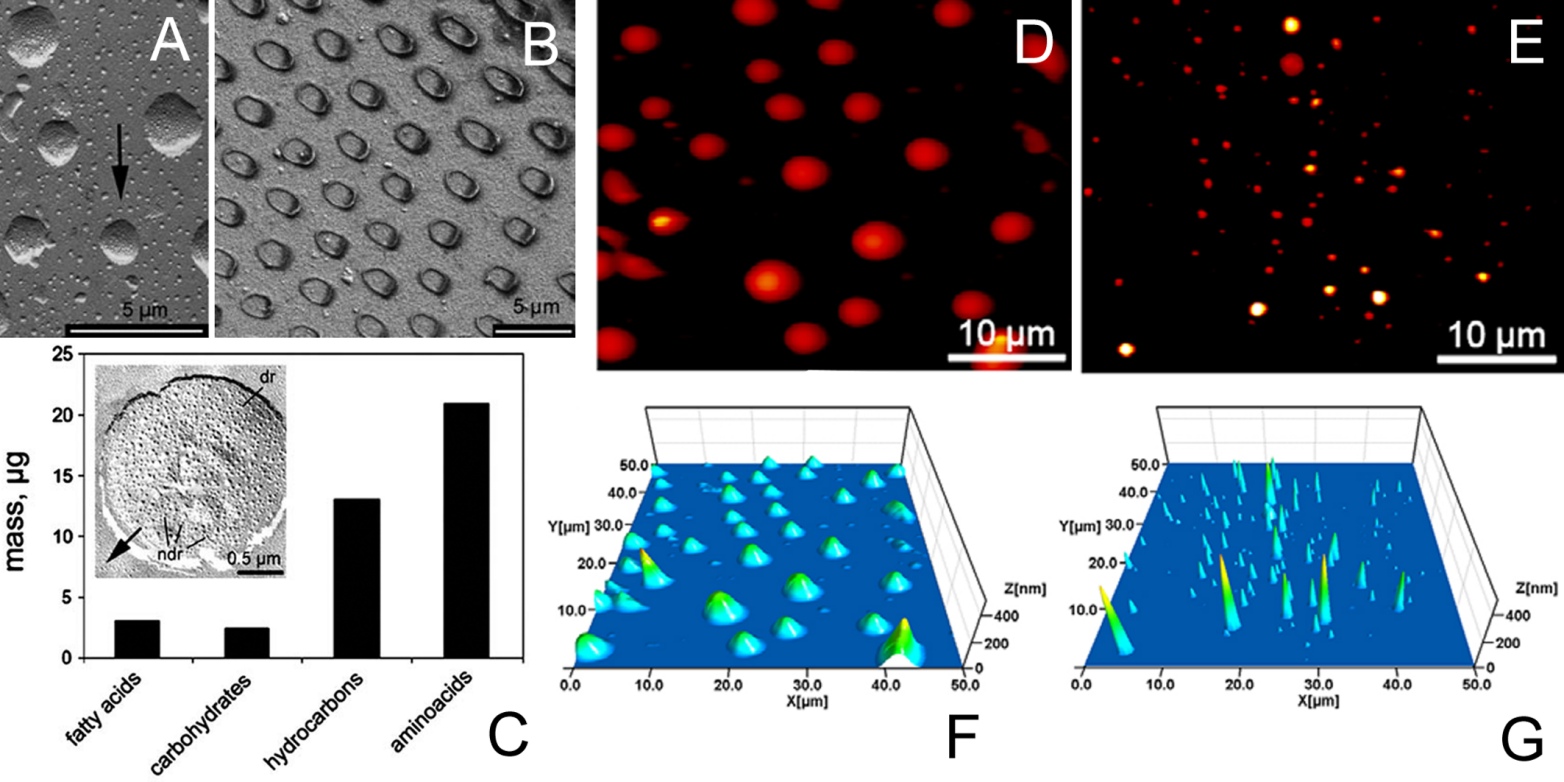
Fluid micro- and nanodrops in animal attachment pads. (A) Carbon–platinum replica of frozen and coated droplets of the fly *Calliphora vicina* in SEM (black arrow indicates the direction of coating). Please note the pattern of nanodrops on the surface of the major droplets (Figure 8A is from [3] and was adapted by permission from Springer Nature from “Attachment devices of insect cuticle” by S. N. Gorb, Copyright 2001 Springer Nature. This content is not subject to CC BY 4.0). (B) Menisci formed around single terminal contact elements of the setae of *C. vicina*. The fly leg was frozen in contact with smooth glass, carefully removed, and the fluid residues were viewed in cryo-SEM (Figure 8B is from [241] and was adapted by permission from Springer Nature from “Biological fibrillar adhesives: functional principles and biomimetic applications. In: Handbook of Adhesion Technology” by S. N. Gorb, Copyright 2011 Springer Nature. This content is not subject to CC BY 4.0.). (C) Chemical composition (absolute concentration of substance groups) of the pad secretion of the smooth euplantulae of *Locusta migratoria* (Figure 8C was adapted from [254], Insect Biochem. Mol. Biol., vol. 32, by W. G. Vötsch; R. Nicholson; Y.–D. Müller; S. Stierhof; S. N. Gorb; U. Schwarz, “Chemical composition of the attachment pad secretion of the locust Locusta migratoria”, pages 1605–1613, Copyright (2002), with permission from Elsevier. This content is not subject to CC BY 4.0). (D–G) Atomic force microscopy (AFM) height images of the footprint droplets of the beetle *Coccinella septempunctata* (D,F) and the fly *Calliphora vicina* (E,G). (D) and (E) share the same colour scale. Brighter pixels correspond to higher z values. (F,G) Three-dimensional impressions of the images shown in D and E, respectively (Figure 8D–G was adapted with permission from [262], © 2012 The Company of Biologists Ltd. This content is not subject to CC BY 4.0).

Fluid is reported to be secreted into the contact area in the smooth pads of cockroaches [154], orthopterans [130,254], aphids [123–124], pentatomid bugs [255–258] and hairy pads of reduviid bugs [111], flies [101–102], coccinellid [80,259], and chrysomelid beetles [260]. Footprints can be observed with the light microscope, especially under phase contrast. The hairy pad secretion was chemically studied mostly in representatives of Coleoptera. It contains a non-volatile, lipid-like substance that can be observed in footprints stained with Sudan black. It has been shown that the pad adhesive secretion of ladybird beetles (Coccinellidae) consists of hydrocarbons and true waxes [80,259], which correspond to the composition of the cuticle coverage. Similar data have been obtained for the chrysomelid beetle *Hemisphaerota cyanea* (Chrysomelidae, Cassidinae) [261]. In smooth insect pads, the pad secretion consists of a water-soluble and a lipid-soluble part [254]. Data obtained from shock-freezing, carbon–platinum coating, and replica preparation show that the secretory droplets contain nanodroplets on their surfaces (Figure 8). These results led authors to suggest that the pad secretion is an emulsion consisting of lipoid nanodroplets dispersed in an aqueous liquid.

**Figure 9:**
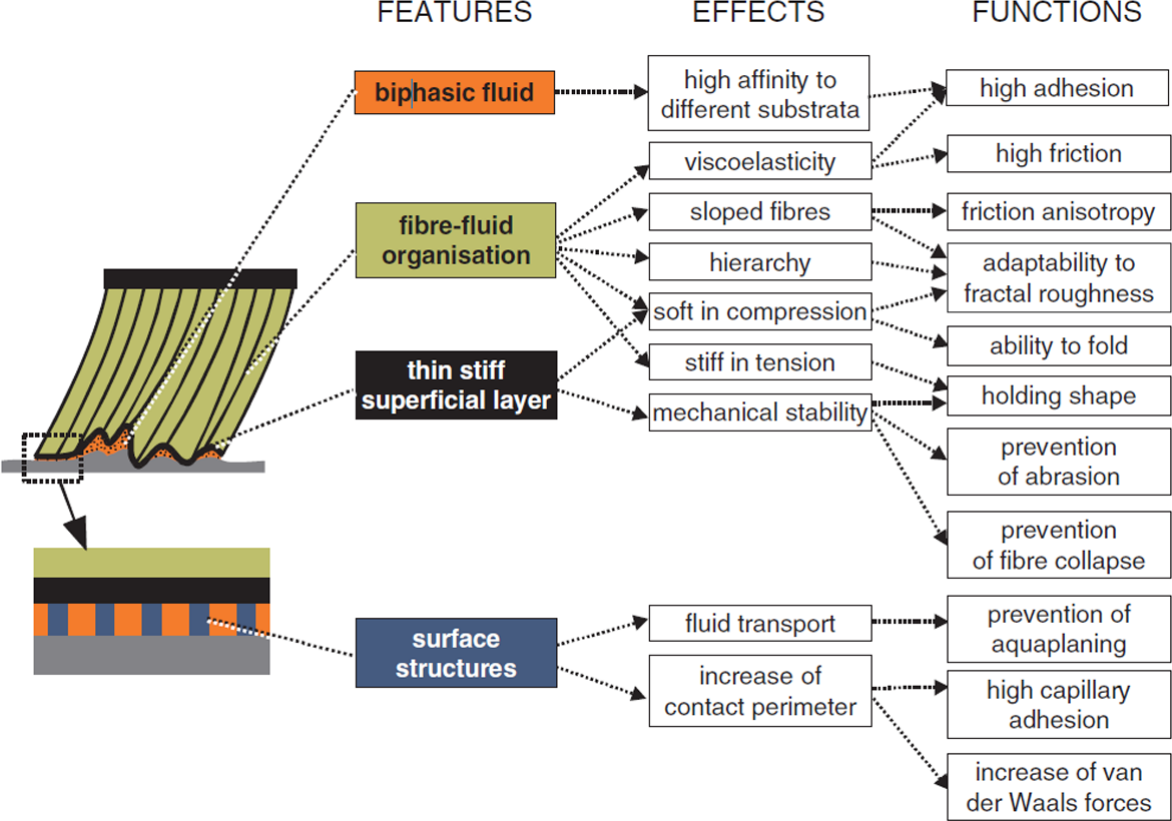
Diagram summarizing structural features of smooth attachment pads that evolved in a convergent matter. Those features are responsible for particular functional effects. Figure 9 was reproduced from [35], “Advances in Insect Physiology: Insect Mechanics and Control.”, 1st Edition, by S. N. Gorb, Chapter “Smooth attachment devices in insects”, pages 81–116, Copyright (2008), with permission from Elsevier. This content is not subject to CC BY 4.0.

The fluid within the smooth pad contributes to the viscoelastic behaviour of the pad since the fluid is able to flow through the gaps between the rods when the pad deforms [129,131]. The fluid, which is released from the smooth pad or from the insect hairy pads into the contact area, may have several functions (Figure 9). It can enhance the contact initialisation due to the capillary forces, which represent long-range interactions. The capillary forces themselves contribute to the adhesion [263]. The fluid can also fill nanoscale gaps on the surface and thus improve contact formation on non-smooth substrata. Since the fluid consists of two phases, it has higher affinity to substrata with various physicochemical properties (hydrophilic and hydrophobic). In other words, the fluid may be a kind of coupling agent, promoting and strengthening the adhesion between otherwise incompatible materials by providing the proximity of contact for intermolecular forces. At a relatively high separation, the contribution of viscous forces to the adhesion and friction are enhanced due to the presence of a thin fluid layer in the contact area [247].

### Significance for biomimetic applications

**Figure 10:**
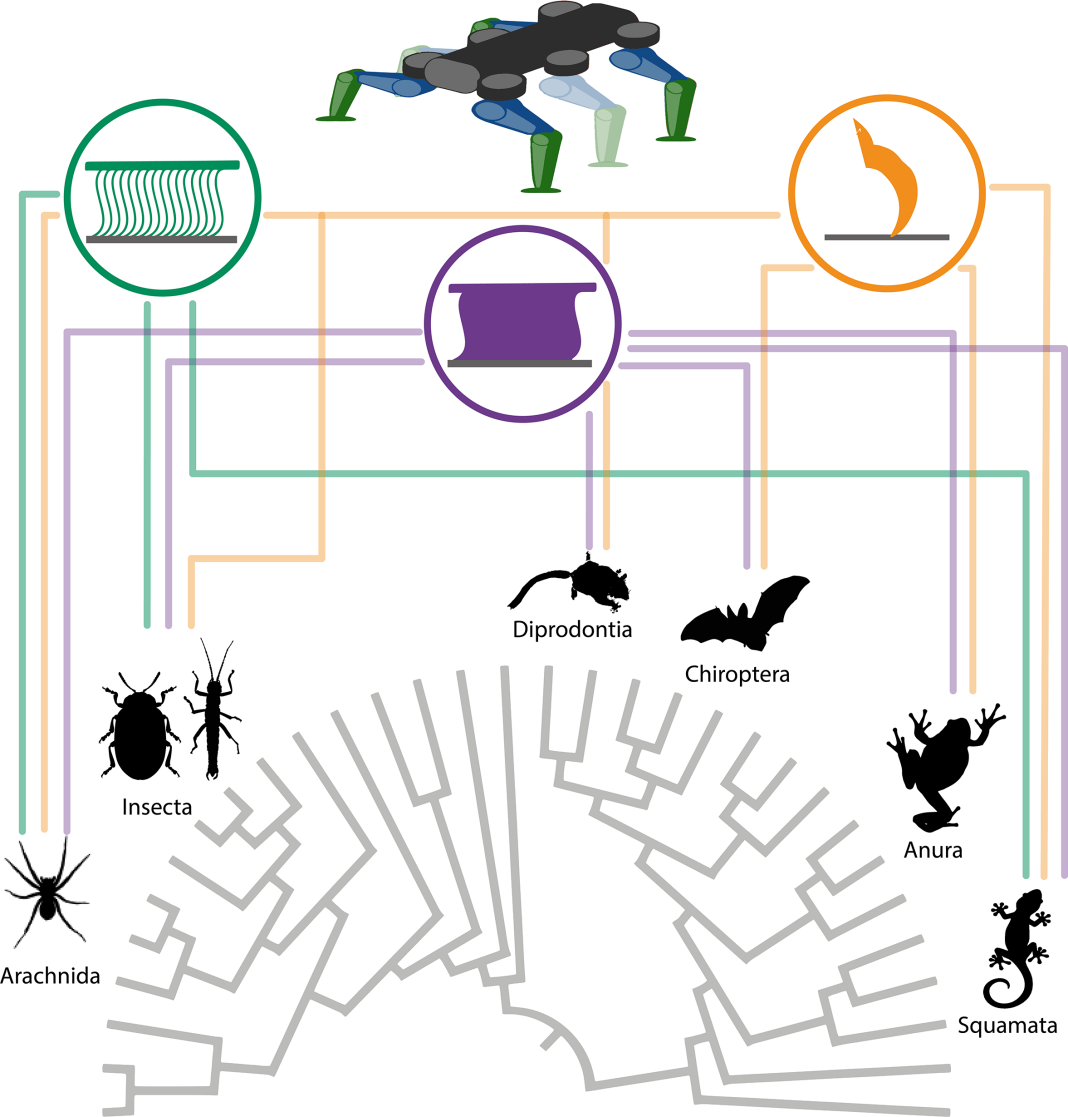
Sources of bioinspiration for attachment systems from the animal tree of life. Shown are the example groups in which attachment systems occur from Figure 1. The functional aspects i) hairy attachment pads (green), ii) smooth attachment pads (purple) and claws (orange), which can inspire the technical application on a four- or six-legged robot is linked by the colour mapping of the groups which include species that represent examples of the mechanism and may serve as a biological source of inspiration. The green and purple icons were adapted with permission from [1], R. G. Beutel et al., “Ultrastructure of attachment specializations of hexapods (Arthropoda): evolutionary patterns inferred from a revised ordinal phylogeny”, J. Zool. Syst. Evol. Res., with permission from John Wiley and Sons. Copyright © 2001 John Wiley & Sons. This content is not subject to CC BY 4.0. The schematic robot was redrawn after [294] (© 2020 Billeschou et al., published by MDPI, distributed under the terms of the Creative Commons Attribution 4.0 International License, https://creativecommons.org/licenses/by/4.0).

Several different approaches to mimic natural adhesive systems have already been published (e.g., reviewed in [264–266]). Different animal groups have been used as templates for designing bioinspired adhesives, ranging from beetles [267–270] to geckos [271–278] and the principles used by these animals were copied to some extent. Examples are the frog-inspired adhesive systems [279–283] and various types of soft gripping devices (reviewed in [284]). The actual strength, however, of natural adhesive systems lies in the fact that the diversity of attachment devices in animals provides a plethora of tools to use as inspiration for engineering. This diversity includes several different solutions for the same problems and can be, therefore, used not only as an inspiration for case examples like geckos, but also as a whole. To isolate the functional concordance between convergent solutions can provide information on the overarching principles that these structures rely on. Since the structure–function relationships discussed above are based on fundamental physical principles and mostly related to the geometry of the structure, they must also hold true for artificial surfaces with a similar geometry. This in turn means that the ideas from biology can be potentially used for engineering applications (Figure 10). Since convergent events are indicators of a kind of optimal solution, or even a single solution developed in the course of biological evolution, broad comparative studies of animal attachment devices can be a great approach for the advancement of biomimetic innovations. For example, further research on the material–function relationship of the attachment pads can be useful for technical applications of artificial attachment systems with either surface-specific use or to provide universal solutions for unpredictable surfaces. The dependence of leg movements and body kinematics can be useful for basic research and applications in the field of robotics [285–288]. In return, robotic systems can provide insights into the regulation and temporal resolution of attachments, which can strengthen experimental results. Furthermore, the characterization of attachment solutions in nature can support the development of bioinspired gripping devices [289–293]. The transfer to technical applications in this context would benefit from the isolation of the collective features of different systems, rather than from emulating a single feature in detail.

## Conclusion

Attachment pads occur in various animal groups. They can be similar across different, phylogenetically unrelated groups, but simultaneously closely related animals can have different attachment systems. As the evolution of such a functional complex is shaped by the surfaces the animals are confronted with, the main driving forces are the physical principles that underlie the process of attachment. Consequently, the constraints influencing the attachment lead to similar solutions in different animals regardless of their position in the tree of life. This review demonstrates the broad range of solutions for the generation of adhesion found in animals and highlights their distribution within the animal kingdom. Similar micro- and nanostructures occur in a convergent manner in different groups, but even different types of attachment pads that differ in the overall morphology rely on similar functional principles. In essence, a high dependency on basic principles leads to a high degree of convergence in animal adhesive pads. Furthermore, the identification of common principles is informative of the most useful solution for attachment problems and biomimetic applications of those can increase our knowledge of the conditions under which these micro- and nanostructures evolved.
